# Supplementary material for: Analysis of PPARγ Signaling Activity in Psoriasis
Source: Int J Mol Sci. 2021 Aug 10;22(16):8603. doi: 10.3390/ijms22168603 (PMC8395241; doi:10.3390/ijms22168603)
Supplement: Supplementary file 1 [file ijms-22-08603-s001.zip › Supplemental materials_Analysis of PPARg signaling activity in psoriasis/Pathway models/Models images and html files/Model of PPARG signaling in psoriasis/11612.html]

TRAF3


# Protein TRAF3

|  |  |
| --- | --- |
| URN | urn:agi-llid:7187 |
| Total Entities | 0 |
| Connectivity | 1126 |
| Name | TRAF3 |
| Description | TNF receptor associated factor 3 |
| Notes | The protein encoded by this gene is a member of the TNF receptor associated factor (TRAF) protein family. TRAF proteins associate with, and mediate the signal transduction from, members of the TNF receptor (TNFR) superfamily. This protein participates in the signal transduction of CD40, a TNFR family member important for the activation of the immune response. This protein is found to be a critical component of the lymphotoxin-beta receptor (LTbetaR) signaling complex, which induces NF-kappaB activation and cell death initiated by LTbeta ligation. Epstein-Barr virus encoded latent infection membrane protein-1 (LMP1) can interact with this and several other members of the TRAF family, which may be essential for the oncogenic effects of LMP1. Several alternatively spliced transcript variants encoding three distinct isoforms have been reported. [provided by RefSeq, Dec 2010] |
| Primary Cell Localization | Cytoplasm |
| ObjectType | Protein |

---

|  |  |
| --- | --- |
| Pathway | NOD-like Receptors |
|  | Antiviral Signaling through Pattern Recognition Receptors |
|  | TLR4 Signaling in Leukocytes |
|  | T-Cell Independent B-Cell Activation |
|  | Immunoglobulin Class-Switch Recombination Activation |
|  | Toll-like Receptors in Sterile Inflammation |
|  | CD16/CD14 Proinflammatory Monocyte Activation |
|  | MAVS in Antiviral Innate Immune Response in Myocarditis |
|  | Dendritic Cells Function in Psoriasis |
|  | Dendritic Cell Activation in Systemic Lupus Erythematosis |
|  | Crohn's Disease |
|  | Dendritic Cell Dysfunction in Crohn's disease |
|  | Follicular Lymphoma |
|  | Proteins Involved in Hodgkin Lymphoma |
|  | Hodgkin Lymphoma |
|  | Mantle Cell Lymphoma |
|  | mTOR/NF-kB/BCR Signaling Disregulation in Mantle Cell Lymphoma |
|  | Splenic Marginal Zone Lymphoma |
|  | Osteoclast Activation in Multiple Myeloma |
|  | Multiple Myeloma |
|  | TNFR/NF-kB Alternative Pathway in Multiple Myeloma Plasma Cells |
|  | Antigen-Presenting Cell Role in Asthma |
|  | B-Cell Mediated IgE Production in Asthma |
|  | Osteoclast Activation in Rheumatoid Arthritis |
|  | HPV E6 and E7 Induced Disruption of TNFR/NF-kB Signaling in Keratinocytes |
|  | Genes with Mutations in Cancer-Associated Sustaining of Proliferative Signaling |
|  | NFKB Canonical Signaling Activation in Cancer |
|  | NFKB Non-Canonical Signaling Activation in Cancer |
|  | Genes with Mutations in Cancer-Associated Evading of Growth Suppressors |
|  | Proteins with Altered Expression in Cancer-Associated Evading of Growth Suppressors |
|  | NFKB Signaling Activation by Blocking of Tumor Suppressors |
|  | CD40 -> Expression Targets in Lymph Node |
|  | NF-kB non-Canonical Signaling |
|  | TNF-alpha/TNFRSF1B Signaling |
|  | NF-kB Canonical Signaling |
|  | pathway 4 |
|  | New Pathway (2) |
|  | Model of PPARG signaling in psoriasis |
|  | sars-cov binding |
|  | sars-cov no binding |
|  | dsRNA induces innate immunity and INFs in alveolar cells |
|  | Dendritic Cell Activation in COVID19 |
|  | 1\_Insufficient activation of immune response in the middle ear epithelium cells in otitis media |
|  | 1\_Defects in response to pathogens in the gut promotes inflammation in Crohn's disease |
|  | 6\_p12I Viral protein promotes T-cell activation in HTLV-1 infection |

---

|  |  |
| --- | --- |
| Group | Genes with Mutations Associated with Splenic Marginal Zone Lymphoma |
|  | Genes with Mutations Associated with Multiple Myeloma |
|  | binding partnetrs for SARSs |
|  | other partners for SARS |

---

|  |  |
| --- | --- |
| MedScan ID | 7187 |

---

|  |  |
| --- | --- |
| LocusLink ID | 7187 |
|  | 22031 |
|  | 362788 |

---

|  |  |
| --- | --- |
| Alias | LMP1-associated protein 1 |
|  | CD40 associated protein I |
|  | CRAF1 |
|  | TRA3\_HUMAN |
|  | CD40BP |
|  | CD40 receptor associated factor I |
|  | TRAFAMN |
|  | CD40 associated protein 1 |
|  | TRAF3 |
|  | IIAE5 |
|  | TNF receptor-associated factor 3 |
|  | OTTMUSP00000041798 |
|  | TRA3 |
|  | tumor necrosis factor receptor associated factor 3 |
|  | CD40 binding protein |
|  | RING-type E3 ubiquitin transferase TRAF3 |
|  | CD40 receptor associated factor 1 |
|  | RNF118 |
|  | CD40-binding protein |
|  | LMP1 associated protein |
|  | TNF receptor-associated factor III |
|  | CRAF-1 |
|  | AI528849 |
|  | CAP I |
|  | LAP I |
|  | LMP1-associated protein |
|  | CAP1 |
|  | LAP1 |
|  | CAP-1 |
|  | amn |
|  | T-BAM |
|  | CD40 receptor-associated factor 1 |
|  | tumour necrosis factor receptor associated factor 3 |

---

|  |  |
| --- | --- |
| EC Number | 2.3.2.27 |
|  | 6.3.2.- |

---

|  |  |
| --- | --- |
| GO ID | 0019901 |
|  | 0019903 |
|  | 0031996 |
|  | 0005164 |
|  | 0031625 |
|  | 0004842 |
|  | 0008270 |
|  | 0035666 |
|  | 0008063 |
|  | 0006915 |
|  | 0045087 |
|  | 0032088 |
|  | 0016579 |
|  | 0016567 |
|  | 0042981 |
|  | 0001817 |
|  | 0050688 |
|  | 0032648 |
|  | 0030162 |
|  | 0007165 |
|  | 0002224 |
|  | 0033209 |
|  | 0035631 |
|  | 0009898 |
|  | 0005829 |
|  | 0005768 |
|  | 0005739 |
|  | 0032991 |
|  | 0046872 |
|  | 0016740 |
|  | 0002376 |
|  | 0005737 |
|  | 0006917 |
|  | 0016874 |
|  | 0032480 |
|  | 0034142 |
|  | 0002756 |
|  | 0005515 |
|  | 0034138 |
|  | 0070740 |
|  | 0018169 |
|  | 0070736 |
|  | 0070735 |
|  | 0070738 |
|  | 0070737 |
|  | 0070739 |
|  | 0004871 |
|  | 0043773 |
|  | 0008766 |
|  | 0043774 |

---

|  |  |
| --- | --- |
| KEGG ID | hsa:7187 |
|  | mmu:22031 |
|  | rno:362788 |

---

|  |  |
| --- | --- |
| Organism | Homo sapiens {Organism urn:agi-taxid:9606} |
|  | Mus musculus {Organism urn:agi-taxid:10090} |
|  | Rattus norvegicus {Organism urn:agi-taxid:10116} |
|  | Homo sapiens |
|  | Mus musculus |
|  | Rattus norvegicus |

---

|  |  |
| --- | --- |
| Mouse chromosome position | 12 60.94 cM |
|  | 12 55.0 cM |

---

|  |  |
| --- | --- |
| OMIM ID | 601896 |
|  | 614849 |

---

|  |  |
| --- | --- |
| Rat chromosome position | 6q32 |

---

|  |  |
| --- | --- |
| Hugo ID | 12033 |
|  | HGNC:12033 |

---

|  |  |
| --- | --- |
| Human chromosome position | 14q32.32 |

---

|  |  |
| --- | --- |
| Swiss-Prot Accession | Q13114 |
|  | A6NHG8 |
|  | Q13114.2 |
|  | Q3UHJ1 |
|  | Q60803 |
|  | Q60803.2 |
|  | D3Z9G0 |
|  | B7Z8C4 |
|  | Q12990 |
|  | Q13076 |
|  | Q13947 |
|  | Q6AZX1 |
|  | Q9UNL1 |
|  | B2RPW3 |
|  | Q62380 |

---

|  |  |
| --- | --- |
| PIR ID | A55960 |
|  | S68467 |
|  | I49272 |

---

|  |  |
| --- | --- |
| GenBank ID | NC\_000014 |
|  | NM\_003300 |
|  | NP\_003291 |
|  | NM\_001199427 |
|  | NP\_001186356 |
|  | NM\_145725 |
|  | NP\_663777 |
|  | NM\_145726 |
|  | NP\_663778 |
|  | XM\_011537117 |
|  | XP\_011535419 |
|  | XM\_011537116 |
|  | XP\_011535418 |
|  | XM\_011537118 |
|  | XP\_011535420 |
|  | XM\_017021620 |
|  | XP\_016877109 |
|  | XM\_017021617 |
|  | XP\_016877106 |
|  | XM\_017021619 |
|  | XP\_016877108 |
|  | XM\_017021618 |
|  | XP\_016877107 |
|  | NG\_027973 |
|  | AL117209 |
|  | AL132801 |
|  | CH471061 |
|  | EAW81795 |
|  | EAW81796 |
|  | EAW81797 |
|  | AF110908 |
|  | AAD29276 |
|  | AK303172 |
|  | BAH13910 |
|  | BC075086 |
|  | AAH75086 |
|  | BC075087 |
|  | AAH75087 |
|  | BQ000015 |
|  | BX247977 |
|  | CAD62311 |
|  | L38509 |
|  | AAA68195 |
|  | U15637 |
|  | AAA56753 |
|  | U19260 |
|  | AAA65732 |
|  | U21092 |
|  | AAC50112 |
|  | Q13114 |
|  | NC\_000078 |
|  | NM\_011632 |
|  | NP\_035762 |
|  | NM\_001286122 |
|  | NP\_001273051 |
|  | XM\_006515802 |
|  | XP\_006515865 |
|  | XM\_017315042 |
|  | XP\_017170531 |
|  | XM\_006515798 |
|  | XP\_006515861 |
|  | XM\_006515800 |
|  | XP\_006515863 |
|  | XM\_006515801 |
|  | XP\_006515864 |
|  | XM\_006515799 |
|  | XP\_006515862 |
|  | NM\_001364364 |
|  | AC122023 |
|  | AC127580 |
|  | AF320615 |
|  | CH466549 |
|  | EDL18642 |
|  | AK134159 |
|  | AK137321 |
|  | AK147304 |
|  | BAE27835 |
|  | AK147362 |
|  | BAE27866 |
|  | AK148231 |
|  | AK217743 |
|  | AW702148 |
|  | BC137634 |
|  | AAI37635 |
|  | BC137635 |
|  | AAI37636 |
|  | BF661650 |
|  | CJ127244 |
|  | U21050 |
|  | AAC52175 |
|  | Q60803 |
|  | NC\_005105 |
|  | XM\_006240596 |
|  | XP\_006240658 |
|  | NM\_001108724 |
|  | NP\_001102194 |
|  | AC\_000074 |
|  | AABR07065583 |
|  | AABR07065584 |
|  | AABR07065585 |
|  | AABR07065586 |
|  | AAHX01046668 |
|  | AAHX01046669 |
|  | AAHX01046670 |
|  | CH474034 |
|  | EDL97481 |
|  | XP\_006240660 |
|  | XP\_006240662 |
|  | XP\_006240661 |
|  | XM\_006720244 |
|  | NP\_001041671 |
|  | XP\_006240657 |
|  | AAHY01103548 |
|  | XP\_006240656 |
|  | XP\_006240659 |
|  | AAHY01103549 |
|  | AMYH02029907 |
|  | AMYH02029908 |
|  | AMYH02029909 |
|  | XM\_006240600 |
|  | NM\_001048206 |
|  | AMYH02029910 |
|  | AC\_000146 |
|  | ABBA01048451 |
|  | ABBA01048450 |
|  | ABBA01048453 |
|  | AABR06046334 |
|  | ABBA01048452 |
|  | AABR06046332 |
|  | AABR06046333 |
|  | AABR06046331 |
|  | AC\_000034 |
|  | ABBA01048454 |
|  | NC\_018925 |
|  | XR\_943512 |
|  | XP\_006720307 |
|  | XM\_006240599 |
|  | XM\_006240598 |
|  | XM\_006240597 |
|  | XM\_006240595 |
|  | XM\_006240594 |

---

|  |  |
| --- | --- |
| Swiss-Prot ID | TRAF3\_HUMAN |
|  | TRAF3\_MOUSE |

---

|  |  |
| --- | --- |
| Cell Localization | Cytoplasm |
|  | Endosome |
|  | Mitochondrion |

---

|  |  |
| --- | --- |
| Ensembl ID | ENSG00000131323 |
|  | ENSP00000445998.1 |
|  | ENST00000539721.5 |
|  | ENSP00000454207.1 |
|  | ENST00000560371.5 |
|  | ENSP00000376500.2 |
|  | ENST00000392745.6 |
|  | ENSP00000328003.4 |
|  | ENST00000347662.8 |
|  | ENSMUSG00000021277 |
|  | ENSMUSP00000112517.1 |
|  | ENSMUST00000117269.7 |
|  | ENSMUSP00000021706.4 |
|  | ENSMUST00000021706.10 |
|  | ENSMUSP00000058361.6 |
|  | ENSMUST00000060274.6 |
|  | ENSRNOG00000008145 |
|  | ENSRNOP00000010906.5 |
|  | ENSRNOT00000010906.6 |
|  | ENSRNOP00000074381.1 |
|  | ENSRNOT00000077770.1 |
|  | ENSRNOP00000010906 |
|  | ENSMUSP00000112517 |
|  | ENSMUST00000021706 |
|  | ENST00000392745 |
|  | ENST00000539721 |
|  | ENSP00000454207 |
|  | ENSP00000445998 |
|  | ENSMUSP00000021706 |
|  | ENSMUSP00000058361 |
|  | ENSP00000376500 |
|  | ENSRNOT00000010906 |
|  | ENSP00000328003 |
|  | ENSMUST00000117269 |
|  | ENST00000347662 |
|  | ENSRNOP00000074381 |
|  | ENSMUST00000060274 |
|  | ENSRNOT00000077770 |
|  | ENST00000560371 |

---

|  |  |
| --- | --- |
| MGI ID | MGI:108041 |
|  | 108041 |

---

|  |  |
| --- | --- |
| RGD ID | 1304633 |

---

|  |  |
| --- | --- |
| Unigene ID | Hs.510528 |
|  | Mm.27431 |
|  | Rn.12033 |

---

|  |  |
| --- | --- |
| Homologene ID | 7981 |

---

|  |  |
| --- | --- |
| IPI ID | IPI00297473 |
|  | IPI00973170 |
|  | IPI00310635 |

---
